# The prevalence of syphilis in HIV-seropositive patients: a retrospective study at the regional hospital in Agadir, Morocco

**DOI:** 10.11604/pamj.2019.33.252.15781

**Published:** 2019-07-25

**Authors:** Mohamed Bourouache, Rachida Mimouni, Mohamed Nejmeddine, Smail Chadli, Fatima Benlmeliani, Jamila Sardi, Mourad Malmoussi, Zineb Ouagari, Maryam El Basbassi, Mohamed Aghrouch

**Affiliations:** 1 Department of Biology, Faculty of Sciences, Ibn Zohr University, Agadir, Morocco; 2 Infectious Diseases Group, Laboratory of Cell Biology and Molecular Genetics, Faculty of Sciences, Ibn Zohr University, Agadir, Morocco; 3 Higher Institute for Nursing Professions and Health Techniques, Agadir, Morocco; 4 Laboratory of Bio-Medical Analysis, Hassan II Regional Hospital, Agadir, Morocco; 5 Department of Infectious Diseases, Hassan II Regional Hospital, Agadir, Morocco

**Keywords:** Syphilis, HIV, AIDS, public health, Agadir, Morocco

## Abstract

**Introduction:**

HIV and syphilis are major public health problems in Morocco. The region of Souss-Massa, south-west of the country, hold more than 24% of HIV seropositive cases registered in Morocco during 2009. The aim of this study is to evaluate the seroprevalence of syphilis among HIV seropositive patients in the region of Souss-Massa, south-west of Morocco.

**Methods:**

To evaluate the seroprevalence of syphilis and neurosyphilis among HIV seropositive patients, we retrospectively investigated the medical records of HIV-infected patients attending the regional hospital located in the city of Agadir, during the period comprised between 2011 and 2016.

**Results:**

The population studied involved 1381 males (49.18%) and 1427 females (50.82%) HIV seropositive patients. Among them, 481 patients were seropositive for syphilis and three cases were diagnosed with neurosyphilis. The sex ratio distribution was 243 male (52.71%) and 218 female (47.29%). The prevalence of syphilis among the studied population was estimated to 16.42% with a slight dominance in male (17.63%) compared to female (15.28%). By contrast, neurosyphilis was only detected in male patients, with a prevalence estimated to 0.11%.

**Conclusion:**

Even if the prevalence of HIV and syphilis is stable in the region of Souss-Massa, the prevalence of syphilis among HIV seropositive patients remained high and correlated positively with that of HIV infection. We did not find a significant difference between the genders, in relation to the prevalence of HIV and syphilis. We concluded that it was essential to continue monitoring the population, in order to improve the prevention and the access to the medical care in the south-west of Morocco.

## Introduction

Syphilis is a sexually transmitted infection (STI), associated with the bacterium *Treponema pallidum* [1]. The vast majority of infections are sexually transmitted [2]. However, the infection might also be transmitted from an infected woman to her newborn child [3]. During pregnancy, the syphilis can lead to spontaneous abortion, congenital deformities, or severe neonatal disease [4-6]. This infection, might cause long-term complications if not treated appropriately [7, 8], continues to be a major health concern in Morocco [9, 10]. The syphilis is a progressive disease, which could be classified according to the degree of severity; from primary stage, to a tertiary stage that leads to a disease of the central nervous system, called neurosyphilis [11]. Each stage of the disease is associated with particular symptoms [12]. The overall incidence of syphilis in the world have increased in recent years [13-15], partially due to its association with HIV infection [16]; in particular high-risk groups of the population, including drug users (IDUs), female sex workers (FSWs) and men who have sex with men (MSM) [17-19]. The HIV is still a common causes of morbidity and mortality around the world, particularly in the developing countries [20, 21]. Interestingly, syphilis itself facilitates HIV infection in several ways and vice versa [22]. In 2009, an analysis of the medical records showed that 24.6% of all HIV seropositive cases registered in the country were from the region of Souss-Massa, in the south-west of Morocco [10]. Previous reports that evaluated the association between HIV and syphilis in Morocco focused mostly on some high risk groups; in particular, female sex workers and men who have sex with men [9, 23]. This led to an overestimation of the prevalence of syphilis in the general population of Souss-Massa. The aim of this study is to establish a more accurate assessment of the prevalence of syphilis in patients tested positive for HIV.

## Methods

**Collecting data:** the department of infectious disease of the regional hospital in Agadir covers all HIV seropositive patients from the whole region of Souss-Massa, in Morocco. The medical records of HIV-infected patients, tested between 2011 and 2016, were examined for the presence of cases of syphilis and neurosyphilis.

**Figure 1:**
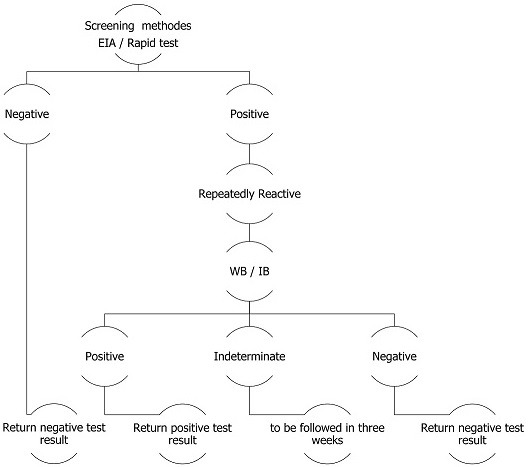
recommended laboratory HIV testing algorithm for serum or plasma specimens

**Screening for HIV:** screening for HIV infection was performed according to the Moroccan Health Ministry recommendations (Figure 1). The diagnostic of HIV infection included a rapid test (Alere Determine^®^HIV-1/2, Alere Inc, Japan) white visual read; qualitative immunoassay for the detection of antibodies to HIV-1 and HIV-2; or the ELISA test (Murex^®^HIV Ag/Ab Combination, Dia-Sorin S.p.A, Saluggia, Italy). In addition, a confirmation test was performed using a Western blot test (MP Diagnostics (MPD) HIV BLOT 2.2, Japan). The interpretations of the tests were performed in accordance with the recommendations of the World Health Organization (WHO), based on the detection of two ENV bands, with or without GAG or POL bands.

**Serological tests for the detection of syphilis:** the serological diagnosis of syphilis was based on a series of two types of serological tests. The first test was a non-treponemal antigen test (VDRL), used for the screening for syphilis in serum or cerebral spinal fluid. The Venereal Disease Research Laboratory test (VDRL; carbon antigen plasmatec laboratory products Ltd, Bridport, UK) allowed the detection of antibodies directed against non-treponemic antigens, called cardiolipins. The second test was a treponemal antigen test TPHA (*Treponema pallidum* hemagglutination assay; immutrep^®^ TPHA, Omega Diagnostics, UK). This test was based on an indirect hemagglutination assay for the detection and titration of antibodies against the causative agent of syphilis, *Treponema pallidum*. The samples that were positive in both tests were then registered as seropositive for syphilis.

**Statistical analysis:** the statistical analysis of the data was performed using the R software, version 3.16. The results were summarized using descriptive statistics. The Welch two sample t-test was used to evaluate the differences between gender (male and female) for both HIV and syphilis prevalence. The Pearson's correlation coefficient was used to assess the correlation between HIV infection and syphilis.

**Ethical considerations:** the data were collected in the hospital register, and the information obtained were kept confidential. The study was approved by the Department of Infectious Disease of the Regional Hospital in Agadir.

## Results

A total of 2808 HIV seropositive patients were included in the present study. The calculated sex ratio was 0.97, for a gender distribution of 1381 males (49.18%) and 1427 females (50.82%). The average annual incidence of HIV infection between 2011 and 2016 was estimated to be about 468 ± 94.41 cases per year. The highest number of new cases was recorded in 2014, with 623 (22.19%) cases. By contrast, the lowest number of new cases was recorded in 2012 with 346 (12.32%) cases (Table 1). We did not detect a statistically significant difference between the male and female seropositive patients (p = 0.788). Among the 2808 HIV seropositive patients, 481 were tested positive for syphilis and 3 cases were diagnosed with neurosyphilis. Syphilis was therefore prevalent in 16.42% in this population, slightly more in males (17.63%) than in females (15.28%). However, this difference was not statistically significant (p = 0,492). The three cases of neurosyphilis detected were all males, placing the prevalence of neurosyphilis in the HIV seropositive patients around 0.11% (Table 2). Statistical analysis of the data showed a significant positive correlation between HIV and syphilis (r = 0.828; p = 0.042).

**Table 1:** Seroprevalence of HIV infection in Souss-Massa between 2011 and 2016

Years	Male N (%)	Female N (%)	Total N (%)
2011	200 (49.1)	207 (50.9)	407 (14.5)
2012	161 (46.5)	185 (53.5)	346 (12.3)
2013	233 (48.2)	250 (51.8)	483 (17.2)
2014	323 (51.8)	300 (48.2)	623 (22.2)
2015	251 (49.9)	252 (50.1)	503 (17.9)
2016	213 (47.8)	233 (52.2)	446 (15.9)
total	1381 (49.2)	1427 (50.8)	2808 (100)

**Table 2:** seroprevalence of syphilis in the HIV seropositive patients between 2011 and 2016

	Syphilis +			Syphilis -		
Years	Male N (%)	Female N (%)	Total N (%)	Male N (%)	Female N (%)	Total N (%)
2011	35 (55.6)	28 (44.4)	63 (15.5)	165 (48.0)	179 (52.0)	344 (84.5)
2012	29 (46.8)	33 (53.2)	62 (17.9)	132 (46.5)	152 (53.5)	284 (84.5)
2013	40 (52.6)	36 (47.4)	76 (15.7)	193 (47.4)	214 (52.6)	407 (84.5)
2014	57 (50.4)	56 (49.6)	113 (18.1)	266 (52.2)	244 (47.8)	510 (84.5)
2015	37 (57.8)	27 (42.2)	64 (12.8)	211 (48.4)	225 (51.6)	436 (84.5)
2016	45 (54.2)	38 (45.8)	83 (18.6)	168 (46.3)	195 (53.7)	363 (84.5)

## Discussion

The region of Souss Massa, south-west of Morocco, is home to 2,677 million inhabitants (according to the latest general population and housing census in 2014), many of them live in Agadir, the capital and the largest city of this region. The department of infectiology within the regional hospital in Agadir provides medical care and drug to almost every HIV seropositive and STIs patients in the region of Souss-Massa [24]. Like everywhere else in the world, in particular in Africa, the sexually transmitted infections (STIs) in Morocco constitute a public health burden. Around 400,000 new cases were registered every year through the public health clinics in the country, but the true burden is believed to be higher, as cases that are not symptomatic and not treated, or which are managed by private health providers or self-treated, are not reported [19]. We reported here the seroprevalence of HIV, syphilis and neurosyphilis among the population in the region of Souss-Massa, then we compared our results with previous reports related to the region of Souss-Massa, and to other regions in Morocco. According to the Health Ministry Department, the region of Souss-Massa is the most affected regions by HIV/AIDS in Morocco. In 2009, this region recorded the highest prevalence of HIV/AIDS in the country (0.9%) [10]. Since then, few studies have been carried out in the area [9, 17, 21, 23-28]; most of them were especially focused on groups that carried a high-risk (female sex workers, men who have sex with men, drug users) [9, 17, 23]. These groups were the main population studied in relation to HIV epidemic in Morocco and elsewhere in the world, with heterosexual sex-worker networks being the largest of the three kinds of high-risk groups, followed by MSM, and then IDUs [10]. These previous studies might have overestimated the influence and the prevalence of HIV and STIs in the general population. The purpose of this study was to examine more accurately the prevalence of syphilis and neurosyphilis in HIV seropositive-patients of SM region for the last six years. New HIV infections were about 468 ± 94.4 cases per year. This incidence was higher than those previously reported in 2012 [17]. The highest number of new HIV cases was recorded in 2014 (Table 1). This might be due to the higher number of screening campaigns that took place in the country in recent years. Indeed, the increased awareness about the disease within the population probably contributed to the decline of stigma towards HIV carriers and some high-risk groups such as MSM and FSWS. In accordance with recent reports [17, 20, 25], the statistical analysis did not show a significant difference in prevalence between male and female populations (p = 0.224). This is contrast to previous studies in Morocco, as well as in most Arab countries, that often showed a gender dominance [10, 17, 21, 25].

We noted a stable incidence of new cases of syphilis, despite the peak incidence in 2014. The prevalence of syphilis between 2011 and 2016 was estimated at 17.13% and did not appear to be affected by the genders (Table 3), consistently with previous report carried out in Agadir and Marrakesh within groups of men who have sex with men [9]. By contrast, it was reported that the prevalence and incidence of active syphilis among women in Morocco was in decline between 1995-2016 [19]. In developing countries, prisons played an important role in HIV and STIs epidemics [29-31]. Prisoners represented a special high-risk group, due to high rates of injected drug users, unprotected sex and the use of non-sterile equipment for tattooing or for shaving [19, 32, 33]. Heijnen *et al* 2016, estimated 496,000 prisoners in MENA, with drug-related offences being a major cause for incarceration [33]. In Morocco, the prevalence of HIV among prisoners is between 0.4% to 0.8%, with higher prevalence in the regions of Souss-Massa-Draa and Marrakech Tensift Al Haouz [32]. But not enough data is available about prisoners in the Souss-Massa region [10, 32], which makes it difficult to assess the contribution of this group in HIV and STIs transmission. The rate of co-infection (Syphilis and HIV) is increasing in North Africa [34-36]; especially, in the group of MSM [37]. A recent report showed that co-infection with HIV and syphilis was estimated to 31.6% in Agadir and 56.4% in Marrakesh [9]. It is well known that the syphilis chancre creates an integument discontinuity, which facilitates the penetration of HIV into the organism [38]. The presence of the virus in the syphilitic ulcers was previously reported [39]. The immunodeficiency state induced by HIV infection [40] can also influence the clinical features and treatment outcome of the syphilis [41]. This was confirmed by the high positive correlation between HIV and syphilis in the MS population (r = 0.828; p = 0.042). The entire of the neurosyphilis cases (3 cases, registered during the year of 2015) were males. The prevalence of neurosyphilis was estimated to 0.11%. This result was in agreement with those reported in 2016 by Fekih *et al.* [42]. The male correlation was probably due to the high frequency of chronic meningo-encephalitis observed in men, which was 4-7 times more common in males than in females [43]. The men who have sex with men (MSM) showed the most exposure to syphilis [2, 14, 44]. Almost 90% of the Moroccan population that engages in intermediate-to-high-risk life style were males [10]. Indeed, 71% of all HIV infections among women were due to an infected spouse [26]. There was no significant difference between male and female groups, for HIV and syphilis prevalence (Table 3). This was probably due to the low rate of MSM in Agadir compared to Marrakech, and to the increased awareness of the general population about sexually transmitted infections (STI) and AIDS.

**Table 3:** summary of the Welch two sample t-test

Variables	x	p-value
**HIV +**		
Male	230.167	0.788
Female	237.833	
**Syphilis +**		
Male	40.500	0.492
Female	36.333	
**Syphilis –**		
Male	189.666	0.6243
Female	201.500	

## Conclusion

Both HIV and syphilis infections reached alarming rates in the region of Souss-Massa in the south-west of Morocco. Despite the peak recorded in 2014, the prevalence of HIV appears to be stable. However, the prevalence of syphilis among HIV patients remained high, and following the same trend as HIV. In order to prevent or to anticipate any further change in the current situation, it is important to keep a permanent scrutiny of the prevalence and incidence in the region of Souss-Massa. This will be essential to provide a better care and to put in place adapted strategies of prevention in Morocco, especially among the most vulnerable in the general population.

### What is known about this topic

- Since 2009, high HIV and syphilis prevalence was reported in the region of Souss-Massa, in Morocco;
- HIV, STIs testing and counseling is a key strategy to reduce sexual risk-taking and control the burden of HIV infection;
- In the region of Souss-Massa, MSM, FSW and prisoners constituted the main high-risk groups carriers of HIV, syphilis and several sexually transmitted infections.

### What this study adds

- The prevalence of syphilis among HIV-infected patients were stable, over the years but remains very high;
- There was a significant correlation between the prevalence of HIV and syphilis infections in the Souss-Massa population;
- Significant efforts will be needed to reduce the prevalence of syphilis and HIV in this region.

## Competing interests

The authors declare no competing interests.
